# Application of Spatial Transcriptomics in Digestive System Tumors

**DOI:** 10.3390/biom15010021

**Published:** 2024-12-27

**Authors:** Bowen Huang, Yingjia Chen, Shuqiang Yuan

**Affiliations:** 1 Department of Gastric Surgery, Sun Yat-sen University Cancer Center, State Key Laboratory of Oncology in South China, Guangdong Provincial Clinical Research Center for Cancer, Guangzhou 510060, China; huangbw1@sysucc.org.cn; 2 Health Science Center, Peking University, Beijing 100191, China

**Keywords:** spatial transcriptomics, tumor microenvironment, spatial heterogeneity, digestive system tumors, inter-cellular interaction

## Abstract

In the field of digestive system tumor research, spatial transcriptomics technologies are used to delve into the spatial structure and the spatial heterogeneity of tumors and to analyze the tumor microenvironment (TME) and the inter-cellular interactions within it by revealing gene expression in tumors. These technologies are also instrumental in the diagnosis, prognosis, and treatment of digestive system tumors. This review provides a concise introduction to spatial transcriptomics and summarizes recent advances, application prospects, and technical challenges of these technologies in digestive system tumor research. This review also discusses the importance of combining spatial transcriptomics with single-cell RNA sequencing (scRNA-seq), artificial intelligence, and machine learning in digestive system cancer research.

## 1. Introduction

Spatial transcriptomics is a newly emerging technology that was declared the method of the year 2020 [1]. In 2016, Ståhl et al. [2] proposed the modern concept of spatial omics for the first time, especially spatial transcriptomics. As a technique used to explore the gene expression within tissues or cells at a spatial dimension [3], spatial transcriptomics integrates high-throughput transcriptome results from tissue slices and locates gene expression profiles back to their original positions, thereby achieving the drawing of spatial transcriptome maps and perform high-throughput analysis of transcripts at different spatial positions in tissues [4].

**Figure 1:**
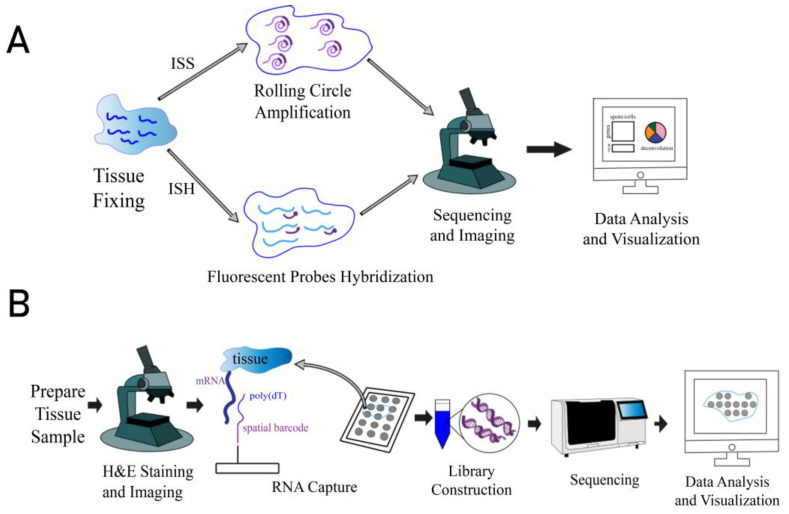
Schematic diagram of three different spatial transcriptomics methods. (**A**) In situ hybridization methods sequence the transcripts within the tissue after rolling circle amplification (RCA), while in situ sequencing methods identify the transcripts by hybridizing with fluorescent probes. (**B**) In sequencing-based methods, transcripts within the tissue are captured by poly(dT) on spatially barcoded microarray slides, after which transcripts are reverse transcribed and sequenced by next-generation sequencing (NGS).

Spatial transcriptomics technologies are classified into imaging-based and sequencing-based methods [5], with the former including in situ hybridization (ISH) and in situ sequencing (ISS) (Figure 1A) [1]. In ISS, RNAs are reverse-transcribed in situ, and the resulting complementary DNA is then hybridized with a specific labeled padlock probe and amplified by rolling circle amplification (RCA) [5]. Finally, RNAs are sequenced in situ [6]. In ISH, the identification of transcripts is achieved by hybridizing a complementary fluorescent probe [5]. The current ISH-based technologies include seqFISH [7], seqFISH+ [8], multiplexed error-robust fluorescence in situ hybridization (MERFISH) [9], etc. Sequencing-based approaches include 10X Genomics Visium, Slide-seq [10], Stereo-seq [11], etc. In 10X Genomics Visium, poly-adenylated RNAs in tissues are captured on spatially barcoded microarray slides [12], then reverse-transcribed and sequenced by next-generation sequencing (NGS) (Figure 1B) [1].

The morbidity of cancer rises rapidly, and cancer is the main cause of death worldwide [13]. The generation, progress, and treatment response of cancer are closely associated with neoplastic cells and non-cancer cells (including stromal cells, immune cells, vascular endothelial cells, etc.) in the tumor microenvironment (TME), as well as the interactions between these cells together with the interactions between these cells and their surrounding environment [14]. It should be emphasized that the immune cells in the TME can infiltrate the tumors and have a decisive impact on the invasive and metastatic ability of tumors, which have a close relationship with the natural course and the treatment outcome of cancer [15].

Spatial transcriptomics can analyze spatial information about various cells in tumor tissues, as well as the information about their gene expression and surrounding environment, which enables us to delve deeper into the spatial structure of tumors and have a clearer understanding of the spatial cellular interactions and the spatial effects in the TME. Nowadays, spatial transcriptomics is widely used in cancer research. It can analyze the spatial heterogeneity of tumors [16], distinguish tumor and non-tumor tissues, identify special spatial areas, such as tumor interface [6], characterize tertiary lymphoid structure in the TME [17], and discover spatially specific prognostic factors in cancer, thus promoting the development of new prevention methods and treatment strategies for cancer.

## 2. Application of Spatial Transcriptomics in Digestive System Tumor Research

### 2.1. Application in Characterizing Tumor Heterogeneity and Uncovering Tumor Cell Subpopulations

Spatial transcriptomics can be used to study tumor heterogeneity. Tumor heterogeneity is an essential characteristic of tumors, including intertumoral and intratumoral heterogeneity. Intertumoral heterogeneity refers to heterogeneity between different patients with tumors of the same histological type, which is induced by genetic variations in the germline, differences between somatic mutation profiles, and environmental factors. Intratumoral heterogeneity describes heterogeneity of tumor cells in an individual patient, including spatial heterogeneity, which manifests as the inhomogeneous distribution of cancer cell subpopulations with genetic diversity across and within disease sites, and temporal heterogeneity, which refers to the dynamic temporal variations in genetic diversity of a single tumor. Because of tumor heterogeneity, various cells in tumor tissues have different characteristics, which result in different responses to cancer treatment. High heterogeneity in tumors is one of the main reasons why cancer is hard to overcome. Therefore, the analysis of tumor heterogeneity plays a significant role in developing effective treatment strategies for cancer [18].

Spatial transcriptomics is widely used to study the heterogeneity of digestive system tumors. For example, Huang H. et al. [19] utilized digital spatial profiling (DSP) to reveal the intratumoral heterogeneity of primary and metastatic esophageal squamous cell carcinoma (ESCC). They found that four immune-related cell types, including B cells, CD8^+^T cells, dendritic cells, and cytotoxic cells, were more distributed in the stromal area of lymph node metastasis subregions than in the stromal area of primary tumor superficial and deep subregions. Yang W. et al. [20] found that autophagy was mainly activated in ductal cells in pancreatic ductal adenocarcinoma (PDAC). In localized squamous cell carcinoma of the anus, Hernandez S. et al. [21] found that for recurrent patients, T cell regulatory-associated protein Foxp3 and MAPK activation markers were mainly expressed in the tumor region, while the immune checkpoint biomarkers were mainly expressed within the TME. Utilizing spatial transcriptomics in conjunction with single-cell RNA sequencing (scRNA-seq), Wang F. et al. [22] found that in colorectal cancer (CRC) primary tumors, plasma cells are more distributed in para-tumor tissues compared with those in tumor tissues. Through spatial transcriptomics analyses of multiple hepatoblastoma-like malignancy samples, Fang J. et al. [23] found that the expression of *Dlk1*, *Epcam*, and *Gpc3*, the embryonal hepatoblastoma stem cell markers, exhibited intertumoral heterogeneity, and the differentiation degree of tumors varied among different patients. Lymph node metastases (LNMs) and tumor deposits (TDs) belong to CRC staging. LNMs are characterized by a clear lymph node structure containing tumor cells, while TDs are aggregates of tumor cells located in the fat near the intestine, surrounding the blood vessels and nerves. Analyses based on spatial transcriptomics revealed significant differences in transcriptome between LNMs and TDs. Genes overexpressed in LNMs were mainly related to immune cells and inflammatory signaling, while genes overexpressed in TDs were mainly associated with cell motility, epithelial–mesenchymal transition (EMT), and matrix remodeling [24].

Spatial transcriptomics is also used to investigate the spatial distribution of cell subpopulations, which can provide us with deeper insights into the complex processes of cancer development and metastasis. Tumors are not homogeneous masses but rather complex structures composed of multiple subpopulations of cells, each with unique characteristics. The spatial arrangement of these subpopulations is thought to be closely linked to their distinct roles in cancer. Chen S. et al. [25] characterized three subpopulations with different metastatic risks in PDAC and analyzed the spatial characteristics of the metastasis-featuring tumor cell subpopulations with the highest metastatic risk through spatial transcriptomics. They found that this subpopulation was mainly distributed in the duct epithelium region, which may contribute to the diagnosis and treatment of PDAC metastasis. In another study about PDAC, Moncada R. et al. [26] integrated the datasets of scRNA-seq and spatial transcriptomics and developed multimodal intersection analysis (MIA), a hypergeometric distribution test method. They identified four ductal subpopulations, including a terminal ductal population, a centroacinar ductal population, a hypoxia ductal population, and antigen-presenting ductal cells. All ductal subpopulations were found to be enriched in ductal tissue, while only the hypoxia ductal population and the terminal ductal population appeared significantly abundant in the cancer area. In a study about ESCC with MIA, Guo W. et al. [27] identified eight subpopulations (C0~C7) in epithelial cells, almost all of which were cancer cells. They found that the stromal areas were the main distribution areas of C4, which was mainly derived from the metastatic sample, and the cancer areas were the main distribution areas of C5 and C7, which were mainly derived from non-metastatic samples.

### 2.2. Application in Studying Tumor Microenvironment

#### 2.2.1. Application in Studying Spatial Distribution Preference of Non-Cancer Cells in Tumor Microenvironment

Spatial transcriptomics has been widely applied to study the spatial distribution of various types of non-cancer cells in the TME. The tumor is composed of malignant cells and their local tissue environment, named TME, where they generate and grow. TME refers to the microenvironment composed of different types of cells, including fibroblasts, endothelial cells, immune cells, and other surrounding cells, and extracellular components, such as extracellular matrix (ECM), cytokines, growth factors, hormones, etc. TME is distributed around cancer cells and receives nourishment from a vascular network [28]. The interaction between tumor cells and TME is involved in the generation and development of cancer and also has an important impact on tumor invasion and metastasis [14].

Fibroblasts and endothelial cells in the TME are the main cellular components of tumor stroma. They have specific preferences for spatial distribution according to research based on spatial transcriptomics [29]. While normal fibroblasts generally suppress the expression of malignant phenotypes in cells [30], cancer-associated fibroblasts (CAFs), which are derived from various mesenchymal cells [31], can promote the production and growth of tumors [32]. In CRC, spatial transcriptomics revealed that *FAP*^+^ fibroblasts and *SPP1*^+^ macrophages colocalized in the tumor tissues and surrounded the tumor epithelial cells, which implies that there was a potential interaction between the two [33]. In ESCC, Guo W. et al. [27] found two subtypes of fibroblasts, including inflammatory CAFs (iCAFs), which were mostly found in stromal areas, and myo-CAFs (myCAFs), which were abundant in cancer areas and stromal areas. Furthermore, among the three subsets (C0, C1, and C2) of endothelial cells, C2 was mainly abundant in the cancer regions, while C0 was mainly distributed in the stromal regions. In diffuse-type gastric cancer, fibroblasts and endothelial cells expressing CCL2 were significantly enriched in the deep layer of gastric cancer tissue [29]. In PDAC, inflammatory fibroblasts, monocytes, and T/natural killer cells were found to be highly enriched in stress-response gene modules [26]. In primary liver cancer (PLC), fibroblasts and endothelial cells were significantly more abundant in the stromal regions [17].

Research based on spatial transcriptomics indicated that the distribution of myeloid cells exhibited spatial differences. In diffuse-type gastric cancer, mast cells were enriched in the normal layers, while dendritic cells were enriched in the deep layers. Monocyte macrophages were divided into five clusters (MM1–MM5), with MM1 and MM3 enriched in the deep layers and MM2 and MM4 enriched in the superficial layers [29]. In PDAC, there were two subpopulations, A and B, of dendritic cells. According to the MIA maps, subpopulation A was most abundant in pancreatic tissue, while subpopulation B was most abundant in ductal tissue [26]. In ESCC, the subpopulation C2 of neutrophils was mainly abundant in the cancer areas of the metastatic sample according to the MIA map [27].

According to the analyses of spatial transcriptomics, immune cells also have a preference for spatial distribution. In ESCC, naïve CD4^+^ T cells, exhausted CD8^+^ T cells, and NK cells were mainly enriched in the cancer areas of the metastatic sample [27]. In oropharyngeal squamous cell carcinoma, spatial transcriptomics revealed the spatial colocalization of immune cells. Nieto P. et al. found that there was a clear correlation between the localization of cytotoxic T cells and regulatory T cells. Furthermore, *SPP1* tumor-associated macrophages (TAMs) and proliferating cancer cells were colocalized, and stromal cells, regulatory T cells, terminally exhausted T cells, and *C1QC* TAMs were also colocalized [34]. Based on the MIA maps, the research found two subpopulations of macrophages in PDAC, including M1 macrophages, which appeared more enriched in the stroma and tumor regions, and M2 macrophages, which were most enriched in the ducts [26]. In PLC, the complete fibrous capsules of tumors affect the distribution of immune cells. For tumors with complete fibrous capsules, the numbers of T cells, B cells, and myeloid cells in the normal area were much higher than that in the tumor area. The numbers of T cells, B cells, NK cells, and myeloid cells in the fibrous capsule area were significantly lower [17]. In CRC, monocytes, NK cells, and epithelial cells were significantly enriched in the tumor area, which uncovered that there was an immune-inflammatory microenvironment in the tumor area. By combining spatial transcriptomics with multidimensional analysis, Peng Z. et al. [35] found that in the mCAFs-enriched cluster, the anti-tumor immune cells and dendritic cells were significantly enriched, while in the iCAFs-enriched cluster, the anti-tumor immune cells, especially NK cells, decreased significantly.

#### 2.2.2. Application in Studying Function of Non-Cancer Cells and Their Interactions

Spatial transcriptomics provides us with further insight into the function of non-cancer cells and their interactions. During the process of tumor growth, invasion, and metastasis, tumor cells come into contact with various types of cells. There may be interactions between tumor cells and these cells, which are closely related to the occurrence and progression of cancer.

In PDAC, there were close spatial connections and interactions between inflammatory fibroblasts and cancer cells expressing stress-response gene modules [26]. CRC was divided into four consensus molecular subtypes (CMS). Valdeolivas A. et al. [36] found that DCN, a proteoglycan secreted by stromal cells, regulated the activity of certain transcription factors by its interaction with receptors, thereby participating in the protective pathway of inhibiting tumor progression in the CMS2 regions with high invasive ability. Moreover, spatial transcriptomics revealed that the colocalization of *FAP*^+^ fibroblasts and *SPP1*^+^ macrophages can activate some pathways that promote the proliferation of connective tissue in CRC [33].

In ESCC, Guo W. et al. [27] annotated the spatial regions that mainly contained metastatic epithelial cells as “Cancer Region 1” and “Stromal Region 1”, and the remaining spatial regions as “Cancer Region 2” and “Stromal Region 2”. Compared to Cancer Region 2, many tumor-related pathways, such as TGF-β pathways, were significantly enriched in Cancer Region 1. EMT and angiogenesis pathways were significantly higher in Stromal Region 2 than in Stromal Region 1. Furthermore, Cancer Region 2 contained a higher proportion of naïve CD4^+^ T cells, gamma delta T cells, M0 macrophages, and eosinophils, while Cancer Region 1 contained a higher proportion of activated NK cells. Most immune cells were mainly distributed in Stromal Region 2, while plasma cells were mainly distributed in Stromal Region 1. In a region with obvious tumor hallmarks in ESCC, EFNB1-EPHB4 ligand–receptor interaction in epithelial cells aberrantly increased, which activated downstream SRC/ERK/AKT signaling, and mediated the cell cycle and EMT of malignant epithelial cells, resulting in the acceleration of cell proliferation and EMT and finally resulting in the progression of ESCC [37].

### 2.3. Application in Studying Function of Tumor Heterogeneity in Treatment Responses

Spatial transcriptomics plays an important role in studying the drug resistance of tumors. Tumor heterogeneity was considered to be the main cause of drug resistance [38]. In GC, Jang E. et al. identified that cluster 2 highly expressed the mesenchymal (pink) module among the malignant cell clusters using spatial transcriptomics. They classified patients who received adjuvant chemotherapy±radiotherapy or surgery alone based on the pink score and observed that patients with a low pink score responded better to adjuvant treatments, while patients with a high pink score did not benefit from adjuvant treatments. However, when patients were stratified based on stromal score or tumor purity, there was no statistically significant difference between the two subgroups, emphasizing that the pink module gene score is more clinically significant than stromal score or cancer purity [39]. 

Spatial transcriptomics uncovered that the specific spatial clusters and intercellular interactions of cell subgroups in tumors can lead to different therapeutic responses. Qi J. et al. [33] observed the colocalization of *FAP*^+^ fibroblasts and *SPP1*^+^ macrophages in CRC. Chemerin secreted by *FAP*^+^ fibroblasts can promote *THBS1*^+^ macrophages to differentiate into *SPP1*^+^ macrophages and the protein synthesized by *SPP1*^+^ macrophages can promote *FAP*^+^ fibroblasts to secrete collagen and MMPs, markers related to the activation of fibroblasts and the remodeling of ECM. Taken together, *FAP*^+^ fibroblasts and *SPP1*^+^ macrophages coordinated to remodel ECM and promoted connective tissue proliferation, preventing the infiltration of lymphocytes into the tumor core and thereby reducing the efficacy of programmed death-ligand 1 (PD-L1) blockade immunotherapy. Therefore, in the clinical treatment of CRC, it is necessary to consider the colocalization of *FAP*^+^ fibroblasts and *SPP1*^+^ macrophages in the TME. Wu Y. et al. [40] also found that there was a certain correlation between the interactions of macrophage subpopulations and the therapeutic response to colorectal cancer liver metastasis.

### 2.4. Application in Tracking Cellular Transitions in Cancer and Elucidating Cancer Evolution

Spatial transcriptomics greatly advances the research of EMT in digestive system tumors. EMT is a reversible cellular process in which polarized epithelial cells achieve the transition from epithelial phenotypes to mesenchymal phenotypes after various molecular and biochemical changes [28]. Notably, EMT involves tumor invasion, metastasis, resistance to therapy, and tumor stemness [41]. In CRC, the sample with an invaded muscular layer exhibited significant enrichment of the EMT-invasion scores in the margin of the right region, while the sample with metastasis into two cancerous nodes showed significant enrichment of the EMT-invasion scores in the margin of the top region, which demonstrated the spatiotemporal relationship between the tumor programs and the EMT status in CRC patients during tumor invasion [42]. In K19-Wnt1/C2mE transgenic (GAN) mice, after injecting the GAN-KP cells into the gastric wall, the GAN-KP-E tumor tissue that formed in the mucosal lamina propria of the gastric epithelium exhibited the characteristics of epithelial-type cancer, while the GAN-KP-S tumor tissue that formed in the submucosa of the gastric epithelium showed the characteristics of mesenchymal-type cancer. In contrast to the nearby GAN-KP-E tumor tissue, the expression of genes associated with hypoxia, angiogenesis, and EMT was higher in the GAN-KP-S tumor tissue. These all indicated that the spatial features of tumors had important effects on intracellular signaling, and the different TMEs resulted in the development of tumors into different EMT states [43].

Spatial transcriptomics is widely used to elucidate the evolution of digestive system tumors. In CRC, the EMT and the migration and invasion of tumors were upregulated in TDs compared to LNMs. Furthermore, TDs had a TME with higher immunosuppressive properties containing more fibroblasts, macrophages, and regulatory T cells. These indicated that there was a phenotype in TDs that was conducive to the progression and immune evasion of CRC [24]. Oral leukoplakia (OLK) is one of the most common precancerous lesions of oral squamous cell carcinoma (OSCC) [44]. In OSCC, Sun L. et al. [45] analyzed tissue samples simultaneously containing early OSCC at the T1 stage (T), OLK with moderate to severe adjacent dysplasia (DN), and normal region (N) with spatial transcriptomics. Fibroblasts were classified into three major subclusters, namely Mesen_CAF, Infla_CAF, and Cycling_CAF. During the initiation process of OSCC, Mesen_CAF showed a gradual increase and played a leading role. Mesen_CAF was mainly distributed throughout entire epithelial regions, which indicated that Mesen_CAF was involved in epithelial cell carcinogenesis, while Infla_CAF was mainly distributed in the lamina regions, which were the connective tissues below the epithelium. Moreover, some immune cells were located near Mesen_CAF in the epithelial regions. During the OSCC initiation process, the monocyte subcluster Mono_INHBA was recruited to the DN region and played a role in the formation of the OLK microenvironment, which led to carcinogenesis. Based on the two characteristics of OLK that separately originate from the upper and lower layers of epithelium, namely hyperkeratosis and dysplasia, the epithelium of N regions and DN regions was divided into two parts. In contrast to the lower layers of N regions and DN regions, the expression of VEGFA signaling, the hallmark of full-blown cancer, was significantly enhanced in the upper layer. This indicated that the upper layer transmitted VEGFA signaling to the proliferating cells in the lower layer, which may improve the proliferation of N regions and DN regions. Collectively, during the OSCC initiation process, VEGFA signaling regulated epithelial interactions through spatial-switching expression.

### 2.5. Clinical Application

Compared to immunohistochemistry, which has long been used by physicians and pathologists for cancer diagnosis, spatial transcriptomics has notable advantages in spatial resolution and throughput, and it can also distinguish cancer subtypes. Therefore, spatial transcriptomics has promising application prospects in the clinical diagnosis of digestive system cancer [6].

Spatial transcriptomics holds great promise in cancer prognosis research. Prognostic factors are closely related to the clinical outcomes of tumors. Three kinds of prognostic factors identified by spatial transcriptomics, including gene markers, gene signatures, and cell subpopulations, have been reported in various digestive system cancers [6]. In CRC, Peng Z. et al. [35] revealed that the proportion of iCAFs in tumor tissues was higher than that in para-carcinoma tissues, which implied that iCAFs may be related to poor prognosis. In PDAC, Croft W. et al. [46] found that the gradients of proximal-to-distant stroma expression may be a prognostic indicator.

Spatial transcriptomics has a promising potential for drug discovery and the development of new therapeutics. Wang et al. [47] used spatial transcriptomics to analyze patients who received neoadjuvant chemotherapy alone or in combination with immunotherapy, that is, in combination with a PD-1 checkpoint inhibitor. Patients receiving chemotherapy showed lower tumor-infiltrating lymphocytes in the tumor regions, while patients receiving combinational therapy showed upregulation of many immune markers in the tumor regions, which indicated that combinational therapy can induce immune infiltration and may have more advantages in the clinical treatment of CRC. These may contribute to the elucidation of immunotherapy mechanisms, the spatial exploration of potential prognostic biomarkers, and the clinical research design for checkpoint inhibitors. Setayesh et al. [48] found that inhibiting matrix formation and foreign antigen recognition in the CD45^+^ cell-enriched region at the tumor margin may facilitate Galectin1 (Gal1) silencing, thereby hindering HCC development. This suggested that Gal1 has broad potential in HCC treatment.

## 3. How to Select a Suitable Spatial Transcriptomics Method

Firstly, when selecting a suitable spatial transcriptomics method, we should consider the experimental objective. Sequencing-based methods are unbiased and do not require prior selection of regions or genes of interest [12], while most imaging-based methods require determining targeted transcripts in advance and prior knowledge to design the corresponding probes [6]. If the experimental objective is to generate a hypothesis, we should choose untargeted methods, such as sequencing-based methods. If the experimental objective is to test a hypothesis, we should choose targeted methods, such as imaging-based methods.

Secondly, the capture efficiency and spatial resolution of spatial transcriptomics methods may affect the analysis of certain genes or tissues with special structures. Sequencing-based methods possess lower RNA capture efficiency [49] and usually cannot achieve single-cell resolution [12], while most imaging-based methods are featured on higher spatial resolution [1], RNA capture efficiency, specificity, and experimental reproducibility [49]. When studying the spatial expression patterns of some genes with significantly lower transcription levels, spatial transcriptomics methods with lower RNA capture efficiency may not be appropriate. Furthermore, due to the limited spatial resolution, it is difficult to study small structures composed of a few cells with methods such as Visium (a representative of sequencing-based methods) [1].

Thirdly, it is necessary to take into account the sample tissue area. The imaging-based methods can analyze a large range of tissue area [5]. However, due to the repetitive imaging processes, when the tissue area is large, imaging-based methods require lengthy imaging time [3]. Differently, the tissue area that Visium can analyze is limited. Therefore, Visium may not be suitable for analyzing tissue with an area that is too small or too large [50].

Fourthly, the quality of mRNAs in the preserved tissues may affect the selection of spatial transcriptomics methods. It can differ between fresh-frozen samples and formalin-fixed paraffin-embedded (FFPE) samples [51]. As time goes on, mRNAs may degrade and become fragmented. We can perform DV200, which detects the proportion of RNA fragments over 200 nucleotides in length to determine if the sample is suitable for spatial transcriptomics [52]. Low DV200 may lead to unsatisfactory sequencing output [1].

**Figure 2:**
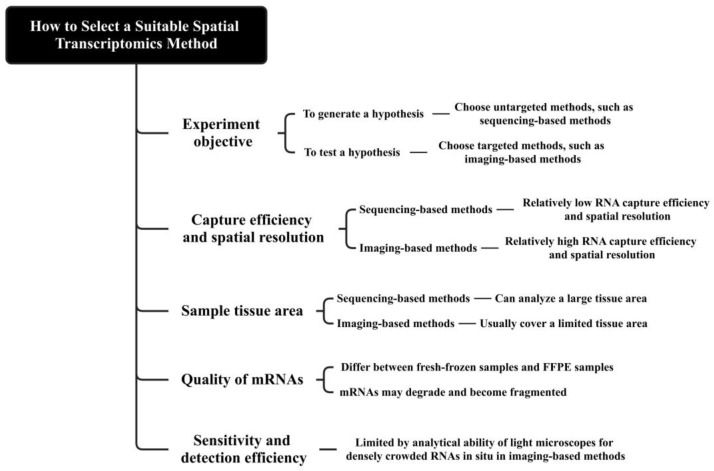
Considerations for selecting a suitable spatial transcriptomics method. When selecting a suitable spatial transcriptomics method, the experiment objective, the capture efficiency and spatial resolution, the sample tissue area, the quality of mRNAs, and the sensitivity and detection efficiency should be taken into consideration.

Finally, it is necessary to consider the sensitivity and the detection efficiency. The analytical ability of light microscopes for densely crowded RNAs in situ limits the sensitivity of imaging-based methods [16]. The sensitivity of ISS is limited by the difficulty in RCA control with the generation of micron-scale amplicons [16]. ISH usually has high sensitivity, such as smFISH with a near 100% detection sensitivity [53,54]. Moreover, the detection efficiency of MERFISH has increased to about 80% recently, which is far higher than the typical detection efficiency (5% to 40%) of scRNA-seq [55,56]. With low sensitivity, microarray-based sequencing methods can only detect nearly 5% of all mRNAs in a cell (Figure 2) [54].

## 4. Data Collection and Analysis in Spatial Transcriptomics Cancer Research

The first analytical assignment executed by spatial transcriptomics is to pre-process the data with technique-specific methods and algorithms, that is, to prepare spatial transcriptomics data for analysis. Typically, performing pre-processing involves converting raw imaging or sequencing data into gene-spot matrices, which are transcript count matrices for each gene through spatial capture regions. Pre-processing data generated from different spatial transcriptomics techniques requires different specific methods [1].

In order to compare the expression of a gene between different spots, data generated from spatial transcriptomics are often normalized, which is to apply statistical transformation to the gene-spot matrix. Normalization is generally achieved by dividing the transcriptomes by the total number of transcripts or performed using regularized negative binomial regression [1,57]. To compare expression between different genes, spatial transcriptomics data are scaled to have the same mean and variance across spots [5].

The spatial data obtained through the above process, including raw gene-spot matrices, normalized data, or accessory data, are subjected to downstream analyses with various utility packages [1]. The first downstream analysis method is deconvolution. Spatial transcriptomics methods without single-cell resolution can capture many cells at each spot, which may contain multiple types of cells. Deconvolution is to estimate the proportions of different cell types in each spot through spatial decomposition algorithms [58]. The common methods are listed in Table 1. The second is to analyze cell–cell and gene–gene interactions, and the common methods are also shown in Table 1. The third is to perform gene imputation. Imputing the missing genes can improve the quality of spatial transcriptomics data. Gene imputation typically involves embedding scRNA-seq and spatial transcriptomics data into a common latent space and integrating information from adjacent scRNA-seq cells for each of the spatial transcriptomics locations [58]. The common gene imputation methods are listed in Table 1. The last is to identify spatially variable genes (SVGs), and the common methods are shown in Table 1. SpatialDE [59] and SPARK [60] have higher computational efficiency than trendseek [61], but their computational complexity and memory requirement are cubic to the number of spatial locations, requiring several days to months of time and dozens to thousands of GB of physical RAM memory to analyze large-scale spatial transcriptomic data [62]. SPARK-X [62] reduces the computational complexity and the physical RAM memory requirement from cubic to linear.

## 5. Challenges and Future Perspectives

### 5.1. Recent Advancements of Spatial Transcriptomics in Cancer Research

Heterogeneity widely exists in genomics, transcriptomics, proteomics, and epigenomics [80]. Spatial transcriptomics has significant advantages in analyzing tumor heterogeneity [81,82]. However, a single omics method can only characterize the tumor heterogeneity from a limited perspective. Nowadays, spatial multi-omics is emerging. For example, parallel detection of transcriptomics and proteomics can be achieved by incorporating fluorophore-conjugated antibodies and oligonucleotide-conjugated antibodies into both in situ and array-based spatial transcriptomics [80]. Currently, spatial multi-omics has been applied in cancer research and has shown promising value [83]. These methods may develop rapidly and greatly deepen our understanding of tumor heterogeneity.

### 5.2. Discussion on Technical Challenges in Spatial Transcriptomics

The ability of optical microscopy to analyze densely crowded RNAs in situ is a major challenge in improving the sensitivity of image-based spatial transcriptomics [16]. In imaging-based methods, when the transcript density is high, the fluorescence signals emitted from these mRNAs usually overlap. Optical crowding restricts the spatial resolution of transcripts and hinders the complete implementation of spatial profiling. MERFISH and seqFISH+ reduce the number of mRNAs that can be detected in a single image to solve this problem. However, this solution requires a large number of fluorescently labeled FISH probes, which means an increase in cost [84], and increases the imaging rounds, thereby reducing analysis efficiency [4]. Therefore, improved technologies with higher sensitivity and advanced analytical methods need to be developed in the future.

Furthermore, there are some limitations in the performance and applicability of spatial transcriptomics. Firstly, compared to scRNA-seq, spatial transcriptomics is unable to capture transcripts with low expression levels [53], which requires improving the RNA capture efficiency of spatial transcriptomics or developing new gene imputation strategies. Secondly, NanoString DSP and 10X Visium are compatible with FFPE samples [85,86,87]. FFPE samples can be stored for a long time, but transcripts in them are usually degraded severely [12]. Therefore, using FFPE samples for spatial transcriptomics may affect the quality of the data. Thirdly, full-length transcripts are helpful for the variant calling of the entire transcriptome and the analysis of the differential expression at the isoform level [88], but most spatial transcriptomics methods can only obtain single-end transcripts. Therefore, spatial transcriptomics is powerless to study immune cell receptor repertoires and alternative splicing events that are both highly associated with tumor research [6].

### 5.3. Future Perspectives of Spatial Transcriptomics in Cancer Research

The occurrence, progression, and metastasis of cancer are usually accompanied by transcriptomic changes. Tumor heterogeneity is closely related to cancer prevention and treatment. The cellular composition of the TME and the communication between these cells play a crucial role in cancer prognosis and treatment response [89].

Integrating spatial transcriptomics and scRNA-seq data can find new cell types and colocalization patterns in the TME, which is helpful to the inference of the interactions between these cells. The deep analysis of tumor heterogeneity and precise characterization of TME structure using spatial transcriptomics will contribute to the discovery of new factors that promote the occurrence of tumors and characterize the malignancy of tumors, which will provide new standards for cancer diagnosis. By utilizing spatial transcriptomics to characterize the different clonal patterns of spatial copy number alterations in different regions of tumors, tumor cells could be distinguished from normal cells and transitional cells, which may bring new hope for early diagnosis of cancer [17,90].

With the emergence and development of artificial intelligence technologies and machine learning technologies, spatial transcriptomics may be able to simplify the diagnostic process and achieve the automation of diagnosis [91,92,93,94]. After being trained with spatial transcriptomics data and annotations on histopathology, spatial transcriptomics acquires the ability to distinguish between normal and diseased regions, divides cancer subregions, and characterizes tumor heterogeneity. This will reduce the errors that may be generated during the manual annotation process and contribute to the selection of different treatment plans for different disease stages or subtypes [4].

## 6. Conclusions

In summary, the sustained development of spatial transcriptomics technologies will open up new avenues for research on digestive system tumors and explore new ideas for the prevention, diagnosis, and treatment of digestive tract cancers.

## Figures and Tables

**Table 1:** A summary of common downstream analyses and the characteristics of their representative methods used to analyze spatial transcriptomics data. DWLS: dampened weighted least squares. NMF: non-negative matrix factorization. NNLS: non-negative least squares. RCTD: robust cell type decomposition. snRNA-seq: single-nucleus RNA-seq. DSTG: deconvoluting spatial transcriptomics data through graph-based convolutional networks. ST: spatial transcriptomics. CCA: canonical correlation analysis. KNN: k-nearest-neighbor. GCN: graph convolutional networks. SVCA: spatial variance component analysis. GCNG: graph convolutional neural networks for genes. gimVI: gene imputation with variational inference. iNMF: integrative non-negative matrix factorization. LIGER: linked inference of genomic experimental relationship. SpaGE: spatial gene enhancement. PRECISE: Patient Response Estimation Corrected by Interpolation of Subspace Embeddings. SVG: spatially variable gene. SPARK: Spatial Pattern Recognition via Kernels. SPARK-X: SPARK-eXpedited.

Common Downstream Analyses	Representative Methods	Characteristics	References
Cell-type deconvolution	spatialDWLS	Utilizes cell type features derived from scRNA-seq data to perform gene signature enrichment and uses DWLS to infer proportions of cell types at each spot.	[58,63,64]
	SPOTlight	Based on a seeded NMF regression algorithm, utilizes cell-type signatures from scRNA-seq data to initialize basis and coefficient matrices, uses NNLS to populate coefficient matrices, and derives proportions of cell types at each spot.	[65]
	RCTD	Uses gene expression profiles for each cell type from scRNA-seq data as input and transcript counts as output to fit a statistical model and leverages maximum-likelihood estimation to infer proportions of cell types at each spot.	[66]
	stereoscope	Based on negative binomial distribution and leverages gene expression profiles from scRNA-seq to infer proportions of cell types at each spot probabilistically.	[67]
	Tangram	Aligns scRNA-seq and snRNA-seq data to spatial transcriptomics data through deep learning, thereby deconvolving low-resolution data into single cells and drawing location maps for different cell types.	[68]
	DSTG	First, generates synthetic pseudo-ST data from scRNA-seq data. Next, performs CCA to incorporate pseudo-ST and real-ST data into a common graph. Then, performs KNN to identify mutual nearest neighbors and construct a link graph of spot mapping. Finally, utilizes a semi-supervised GCN to explain the compositions of cell types at each spot based on the link graph.	[69]
Cell–cell interactions and gene–gene interactions	MISTy	Dissects contribution of different predictor markers to prediction of target marker expression in a specific view and identifies potential interactions between target marker and predictor markers.	[58]
	SVCA	Decomposes variation of gene expression into intrinsic effects, environmental effects, and cell–cell interaction effects and utilizes a gradient-based optimizer to calculate proportion of variance attributable to cell–cell interaction components through maximum likelihood.	[70]
	ProximID	Based on physical cell interaction and scRNA-seq and used to infer cell–cell interactions.	[71]
	GCNG	Encodes position of cells and expression of gene pairs in these cells into two matrices separately as inputs and leverages two graph convolutional layers and a sigmoid function output layer to infer gene–gene interactions.	[72]
	Squidpy	Based on Python, dissects spatial omics data and detects cell–cell interactions mediated by ligand–receptor interactions between identified cell clusters using CellPhoneDB.	[73]
Gene imputation	gimVI	Integrates scRNA-seq and spatial transcriptomic data to impute missing genes.	[58]
	Tangram	Takes scRNA-seq, snRNA-seq, and spatial transcriptomics data as inputs, rearranges scRNA-seq and snRNA-seq profiles in space, and obtains new spatial data containing all genes at single-cell resolution and with spatial position.	[68]
	stPlus	Based on an auto-encoder with a loss function, conducts weighted KNN to perform joint embedding projection and leverages scRNA-seq profiles to achieve accurate prediction for expression of unmeasured genes and effective imputation for measured genes.	[74]
	Harmony	Leverages iterations of maximum diversity clustering and mixture-model-based linear batch correction to project cells into a shared embedding with reduced dimension, thereby embedding scRNA-seq and spatial transcriptomics data into a common latent space and using KNN imputation to infer spatial expression and localization of unmeasured genes.	[75]
	LIGER	Utilizes iNMF to learn a low-dimensional space, integrates scRNA-seq and spatial transcriptomics data to assign spatial positions to cell clusters, and improves resolution for detecting cell clusters from in situ data.	[76,77]
	SpaGE	Based on domain adaptation using PRECISE, integrates scRNA-seq and spatial transcriptomics datasets, corrects differences in sensitivity of transcript detection, and performs KNN to predict spatial expression of unmeasured genes.	[78,79]
SVGs identification	trendseek	Based on marked point theory, conducts pairwise analyses on points as a function of distance between points to evaluate whether there is a significant dependency between spatial distributions of points (represent spatial positions of cells or regions) and their related marks (represent expression levels), and identifies genes with significant spatial expression trends.	[61]
	SpatialDE	Based on Gaussian process regression, decomposes expression variability of each gene into a spatial component (modeled as a spatial variance term that parametrizes gene expression covariance by pairwise spatial distances among locations) and a non-spatial component (modeled as a noise term) and compares full model to a model without spatial variance component to identify significant SVGs.	[59]
	SPARK	Based on a generalized linear spatial model; enables analyzing tens of thousands of genes across tens of thousands of spatial positions.	[60]
	SPARK-X	Based on a robust covariance test framework; enables including various spatial kernels for non-parametric spatial modeling of large-scale sparse spatial transcriptomic data.	[62]

## Data Availability

Data sharing is not applicable.
